# Identification of immune microenvironment subtypes and clinical risk biomarkers for osteoarthritis based on a machine learning model

**DOI:** 10.3389/fmolb.2024.1376793

**Published:** 2024-10-17

**Authors:** Bao Li, Yang Shen, Songbo Liu, Hong Yuan, Ming Liu, Haokun Li, Tonghe Zhang, Shuyuan Du, Xinwei Liu

**Affiliations:** Department of Orthopedics, General Hospital of Northern Theater Command, Shenyang, China

**Keywords:** osteoarthritis, biomarker, machine learning, immune microenvironment, early diagnosis

## Abstract

**Background:**

Osteoarthritis (OA) is a degenerative disease with a high incidence worldwide. Most affected patients do not exhibit obvious discomfort symptoms or imaging findings until OA progresses, leading to irreversible destruction of articular cartilage and bone. Therefore, developing new diagnostic biomarkers that can reflect articular cartilage injury is crucial for the early diagnosis of OA. This study aims to explore biomarkers related to the immune microenvironment of OA, providing a new research direction for the early diagnosis and identification of risk factors for OA.

**Methods:**

We screened and downloaded relevant data from the Gene Expression Omnibus (GEO) database, and the immune microenvironment-related genes (Imr-DEGs) were identified using the ImmPort data set by combining weighted coexpression analysis (WGCNA). Functional enrichment of GO and Kyoto Encyclopedia of Genes and Genomes (KEGG) were conducted to explore the correlation of Imr-DEGs. A random forest machine learning model was constructed to analyze the characteristic genes of OA, and the diagnostic significance was determined by the Receiver Operating Characteristic Curve (ROC) curve, with external datasets used to verify the diagnostic ability. Different immune subtypes of OA were identified by unsupervised clustering, and the function of these subtypes was analyzed by gene set enrichment analysis (GSVA). The Drug-Gene Interaction Database was used to explore the relationship between characteristic genes and drugs.

**Results:**

Single sample gene set enrichment analysis (ssGSEA) revealed that 16 of 28 immune cell subsets in the dataset significantly differed between OA and normal groups. There were 26 Imr-DEGs identified by WGCNA, showing that functional enrichment was related to immune response. Using the random forest machine learning model algorithm, nine characteristic genes were obtained: *BLNK* (AUC = 0.809), *CCL18* (AUC = 0.692), *CD74* (AUC = 0.794), *CSF1R* (AUC = 0.835), *RAC2* (AUC = 0.792), *INSR* (AUC = 0.765), *IL11* (AUC = 0.662), *IL18* (AUC = 0.699), and *TLR7* (AUC = 0.807). A nomogram was constructed to predict the occurrence and development of OA, and the calibration curve confirmed the accuracy of these 9 genes in OA diagnosis.

**Conclusion:**

This study identified characteristic genes related to the immune microenvironment in OA, providing new insight into the risk factors of OA.

## 1 Introduction

Osteoarthritis (OA) is a degenerative disease with a high incidence worldwide. Patients with OA often experience chronic joint pain, swelling, deformity, and discomfort. In the late stage, they may suffer from severe pain or joint stiffness, sometimes leading to a loss of mobility (Allen et al., 2022). This condition subsequently results in a severe economic burden on families, the healthcare system, and society (Quicke et al., 2022). However, the condition progresses and worsens in most patients before joint discomfort or significant imaging changes occur, leaving them in a subclinical stage for a long time. Although there are no obvious manifestations of OA in the subclinical stage, the pathological changes in articular cartilage are irreversible, as the cartilage has a limited ability to repair itself due to the lack of blood vessels, nerves, and lymphoid tissue (Abramoff and Caldera, 2020). Current treatment strategies for OA are limited to reducing symptoms and controlling inflammation, while advanced patients often require joint replacement surgery (Seed et al., 1995). Therefore, developing new diagnostic biomarkers that can reflect articular cartilage injury is crucial for early diagnosis.

In recent years, many scholars have focused on the role of the immune microenvironment in OA development (Quicke et al., 2022; Zhao F. et al., 2023; Nedunchezhiyan et al., 2022). A significant presence of macrophages, neutrophils, and lymphocytes has been identified in the synovial tissue of joints affected by OA. These cells stimulate chondrocytes to produce matrix-degrading enzymes by releasing large amounts of cytokines, which accelerate cartilage destruction and aggravate the chronic pathological process of OA to some extent (Wood et al., 2022; Luo et al., 2023). In addition, various other immune cells, including monocytes, B cells, T cells, NK cells, dendritic cells, and others, interact to drive the pathological changes in cartilage (Sebastian et al., 2022; Woodell May and Sommerfeld, 2020). Interestingly, immune cells also actively participate in articular cartilage regeneration and repair. During cartilage repair, immune cells secrete anti-inflammatory factors that can inhibit inflammation and promote cartilage repair (Li M. et al., 2022). Given the complex immune microenvironment and the numerous immune cells involved in the pathological process of OA, it is necessary to develop a systematic method to evaluate the close relationship of the immune microenvironment in pathogenesis, explore immune-related biomarkers, and predict the risk of OA.

Some scholars have attempted to identify biomarkers from cartilage or subchondral tissue samples of clinical patients; however, these methods are invasive and may lead to iatrogenic joint trauma and inevitable damage to patients (Ge et al., 2021). In recent years, bioinformatics has been widely used for the identification of biomarkers in various diseases and the exploration of potential molecular mechanisms (Bhattacharya et al., 2018). Using bioinformatics and machine learning to analyze immune-related differentially expressed genes (Imr-DEGs) and explore the characteristics of different epidemic subtypes of diseases is a multidisciplinary approach to studying the molecular mechanism of diseases (Orlov et al., 2021). An increasing number of genes have been identified in OA and are also used to study other types of diseases, such as rheumatoid arthritis (Ge et al., 2021; Zhang et al., 2021). While the infiltration of immune cells is essential for the development of OA, only a few studies used various methods to explore the relationship.

In this study, we analyzed multiple Gene Expression Omnibus (GEO) datasets to identify DEGs in OA and normal tissues. We evaluated the immunophenotyping model of OA using single-sample gene set enrichment analysis (ssGSEA), the least absolute shrinkage and selection operator (LASSO), and weighted gene co-expression network analysis (WGCNA). Additionally, we constructed a machine learning model and developed a nomogram-based tool for predicting OA occurrence. Differences in immune characteristics among various immune subtypes were identified, and we explored the relationship between feature genes and potential drug interactions. This study stands out for its comprehensive integration of Imr-DEGs with machine learning and bioinformatics to develop a robust model for OA diagnosis. Unlike previous research that focused on individual immune cells or pathways, our approach systematically evaluates the complex immune microenvironment in OA, identifies distinct immune subtypes, and links these findings to potential therapeutic targets. This methodology offers new biological insights and paves the way for more precise diagnostic tools and personalized treatment strategies for OA.

## 2 Methods

### 2.1 Public data download and processing

Transcriptome expression profile data were downloaded from the Gene Expression Omnibus database (GEO, https://www.ncbi.nlm.nih.gov/geo/). The GEO datasets were selected based on the inclusion of only untreated samples with OA and control groups. The selected datasets include GSE178557, GSE169077, GSE117999, GSE206848, GSE55235, GSE55457, GSE98918, GSE82107. Only OA and normal samples were considered in the analysis, excluding rheumatoid arthritis samples found in GSE206848, GSE55235, and GSE55457. For training purposes, we used synovium samples from GSE206848 (7 OA: 7 normal), GSE55235 (10 OA: 10 normal), GSE55457 (10 OA: 10 normal), GSE98918 (12 OA: 12 normal), and GSE82107 (10 OA: 7 normal). Validation was performed using cartilage samples from GSE178557 (4 OA: 4 normal), GSE169077 (5 OA: 5 normal), and GSE117999 (12 OA: 12 normal). This approach ensures the robustness of the identified OA biomarkers across different tissue types. The data sample size, sample type, and log2 processing details are summarized in Table 1.

**TABLE 1:** Data sample size and sample type.

ID	Sample type	Sample size	Log2 processing	Dataset type
GSE178557	Osteoarthritis: Normal cartilage	4: 4		Validation
GSE169077	Osteoarthritis: Normal cartilage	5: 5	Yes	Validation
GSE117999	Osteoarthritis: Normal cartilage	12: 12		Validation
GSE206848	Osteoarthritis: Normal synovium	7: 7	Yes	Training
GSE55235	Osteoarthritis: Normal synovium	10: 10	Yes	Training
GSE55457	Osteoarthritis: Normal synovium	10: 10	Yes	Training
GSE98918	Osteoarthritis: Normal synovium	12: 12		Training
GSE82107	Osteoarthritis: Normal synovium	10: 7	Yes	Training

Note: GSE206848, GSE55235, and GSE55457 also include some rheumatoid arthritis samples, and only osteoarthritis and normal samples are considered in the analysis. External data set verification uses cartilage samples, and the results are consistent with synovial samples, which indicates that key genes can be used as markers for the diagnosis of osteoarthritis.

During the initial data processing phase, we applied the “NormalizeBetweenArrays” function from the “limma” package within each dataset to correct for batch effects, aiming to minimize non-biological variability caused by experimental conditions. This normalization step, including log2 transformation for datasets GSE169077, GSE206848, GSE55235, GSE55457, and GSE82107, ensured that data from different batches were comparable without altering relative expression differences between samples.

To address the variation in probe numbers across platforms, probes from each dataset were mapped to corresponding gene symbols based on chip annotation information. For genes with multiple corresponding probes, the median expression value was used as the final gene expression level. This approach allowed for consistent comparison of gene expression across datasets. Only genes with corresponding symbols across all datasets were retained to minimize the impact of differing probe numbers.

After preprocessing and gene symbol alignment, the datasets of GSE206848, GSE55235, GSE55457, GSE98918, and GSE82107 were merged. The “ComBat” function from the “sva” package was then used to remove any remaining batch effects by modeling them as random effects and adjusting the data accordingly. Following this, we conducted principal component analysis (PCA) to confirm that batch effects had been effectively addressed, ensuring the data was ready for subsequent differential analysis. After removing batch effects, the inherent biological variations between the samples were preserved for accurate differential analysis.

### 2.2 Immune infiltrating cells and immune score evaluation

ssGSEA was used to evaluate the enrichment fraction of 28 immune cell subtypes (Jin et al., 2023). The “GSVA” package was used to evaluate the enrichment of different cells across samples by transforming the gene expression matrix into a gene set expression matrix between samples. The Wilcox test was employed to evaluate differences in immune cell abundance between the two groups. The R package “estimate” was used to assess the level of immune infiltration.

### 2.3 Identification of characteristic immune cells

In this study, the R package “glmnet” was used to construct the LASSO regression model. The optimal variables were determined from the immune cells, with the data used as the merged dataset. To avoid model instability, 10,000 iterations and 10-fold cross-validation were employed. The minimum criterion was used to determine the optimal penalty parameter. The immune cell subsets with non-zero coefficients were considered the optimal variables and were applied to the following analysis.

### 2.4 WGCNA

The WGCNA network was constructed using the “WGCNA” R package. The optimal immune cells identified earlier were used as phenotypes to identify the gene modules related to immune cell subtypes. Patient samples from the merged dataset were selected, and the top 50% of the variance was screened as input data. The outliers were removed, and the optimal soft threshold was determined based on the scale-free topology criterion. The transformation between the weighted adjacency matrix and the topological overlap matrix was performed. The hierarchical clustering tree method was used to identify modules with more than 30 genes, with the merging distance for similar modules set to 0.25. Each module was displayed in a random color. Finally, the corresponding genes of these modules, identified through phenotypic correlation screening, were used for further analysis.

### 2.5 Evaluation of genes related to differential immunity

The Immunology Database and Analysis Portal database (ImmPort; https://www.immport.org/home) was used to download the immune-related gene set, and the R package “limma” was used to analyze the differences between disease and normal groups. The screening criteria for differential genes were |log2FC| > 0.5 and a *p*-value <0.05. The genes from the three modules of the selected immune gene set were intersected with differential genes and ImmPort genes, and then combined to identify the differential genes related to the immune microenvironment (Imr-DEGs).

### 2.6 Protein interaction and functional enrichment analysis

The STRING database (https://cn.string-db.org/) was used to explore the interaction of the Imr-DEGs, and Cytoscape software was used for visualization. The R package “clusterProfiler” was employed to perform GO and KEGG functional enrichment of these differential immune genes. The *p*-value was corrected using the Benjamini–Hochberg method, and the correlation enrichment results after correction are shown.

### 2.7 Machine learning model construction and optimal model selection

According to the grouping information of cases and controls, the merged dataset was randomly divided into training and verification sets in a 7:3 ratio to maximize model learning while ensuring reliable performance evaluation (Jones et al., 2023). Based on the expression profiles of the Imr-DEGs, random forest (RF), support vector machine (SVM), and logical regression (LR) machine learning models were constructed using the R package “randomForest,” “e1071,” and “caret,” respectively. The accuracy of the model was tested on both the training set and verification set and was illustrated by the ROC curve. The optimal model was determined based on the Area Under the Curve (AUC) value. The optimal model then screened the characteristic genes, and we tested the ability of these characteristic genes to distinguish between disease and control.

### 2.8 Key feature verification and nomogram construction

The diagnostic ability of the characteristic genes was verified using external datasets GSE178557, GSE169077, and GSE117999. The R package “rms” was used to fit the characteristic genes to establish a nomogram, and the “regplot” package was used to visualize the nomogram. The accuracy of the nomogram was assessed using the calibration curve.

### 2.9 Recognition of different immune subtypes by unsupervised clustering

Based on the characteristic gene expression profiles of patients with combined data, the R package “ConsensusClusterPlus” was used to identify different immune subtypes. The clustering algorithm used partitioning around medoid (PAM) with 500 iterations, using 80% of the samples each time. The Euclidean distance was selected as the clustering method, with the corresponding parameters set as: reps = 500, pItem = 0.8, ClusterAlg = “pam”, distance = “euclidean”. The “Rtsne” package was used to visualize the distribution of immune subtypes.

### 2.10 Analysis of the characteristics of different immune subtypes

Functional enrichment of different immune subtypes was assessed using the R package “GSVA”. The gene sets “c2.cp.kegg.symbols” and “c5.go.symbols” were extracted from the R package “msigdbr”. The Drug-Gene Interaction Database (DGIdb, www.dgidb.org) was used to explore the relationship between characteristic genes and drugs, and the interaction network diagram was visualized by Cytoscape software.

### 2.11 Statistical analysis

All statistical analyses were performed using R (v4. 0). The R packages “FactoMineR” and “factoextra” were used for PCA and visualization. The heat map was visualized using the “pheatmap” package, the “VennDiagram” package was used for Venn diagram visualization, and the “pROC” package was used for ROC curve visualization. Unless otherwise noted, results were visualized using the “ggplot2” or “plot” packages. Pearson’s correlation method was used for correlation analysis. The Wilcox test was used to compare differences between two groups, and the ANOVA test was used to assess differences among multiple groups. A *p*-value of <0.05 was considered statistically significant. ∗represented *p* < 0.05, ∗∗represented *p* < 0.01, ∗∗∗represented *p* < 0.001, and “ns” indicated no statistical significance.

## 3 Results

### 3.1 Immune infiltration in patients with OA

**FIGURE 1:**
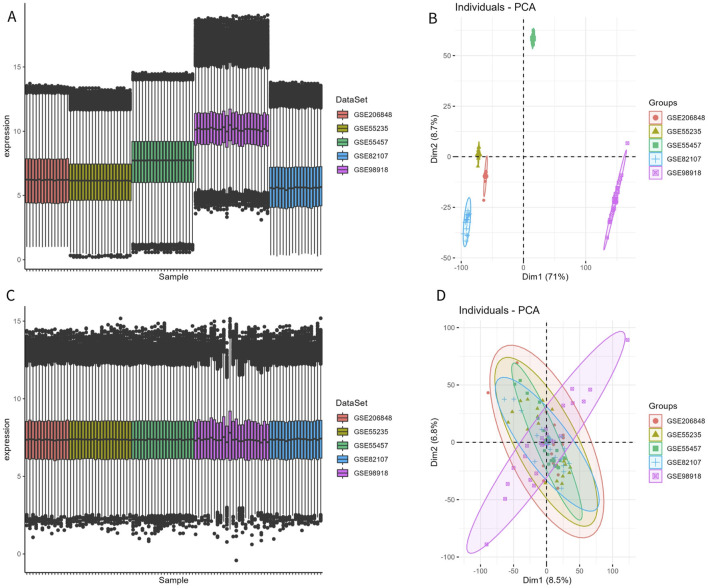
Removal of batch effect. **(A, B)** Expression box plot and principal component analysis (PCA) of different datasets before batch effect removal. **(C, D)** Expression box plot and PCA of different datasets after batch effect removal.

The disease and control samples from the GSE206848, GSE55235, GSE55457, GSE98918, and GSE82107 datasets were combined, yielding 49 patients with OA and 46 control samples. Before removing the batch effect, the sample boxplot of the different datasets was irregular, with an obvious separation between the OA and control groups, showing different clustering patterns (Figures 1A, B). After removing the batch effect, the sample boxplot became uniform, and the different datasets clustered into the same pattern (Figures 1C, D).

**FIGURE 2:**
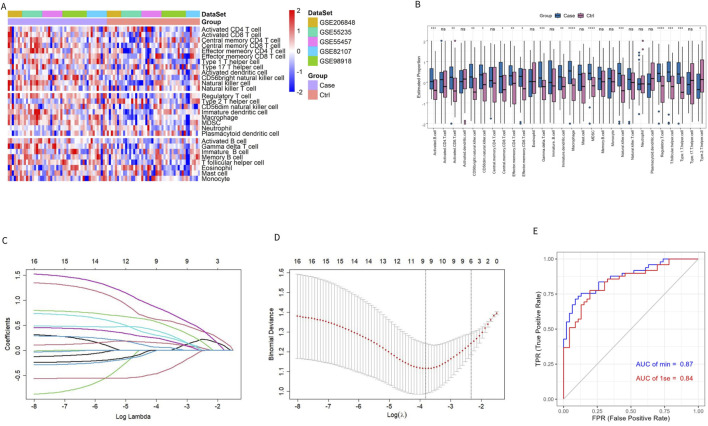
Evaluation of immune cell infiltration in patients with OA and controls. **(A, B)** Heatmap and box plot of ssGSEA enrichment score for 28 types of immune cells in the combined dataset. **(C)** Distribution of the LASSO coefficient of 16 immune cell subtypes. **(D)** Ten-fold cross-validation LASSO regression analysis; the dotted line represents the model’s minimum lambda (λ) and optimal λ. **(E)** The model’s ROC curve based on different λ values.

To characterize the immune difference between patients with OA and controls, the enrichment scores of 28 immune cell subtypes in the combined datasets were compared. The results showed significant differences in 16 immune cell subtypes between the two groups (Figures 2A, B). In addition to Type 2 T helper (Th2) cells, 15 significantly different immune cells infiltrated the OA group. More information on the *p*-value of the immune cell comparisons between groups in the combined dataset is provided in Supplementary Table S1.

The LASSO regression algorithm further identified the characteristic immune cells associated with OA. The LASSO coefficient distribution map displayed the different coefficient distributions of 16 genes (Figure 2C). Additionally, the ROC curve showed that the model accuracy was higher (AUC = 0.87) when the lambda was minimal (Figures 2D, E). The minimum corresponding value of Lambda was 9, resulting in the final determination of 9 variables with non-zero coefficients from the 16 immune cells, corresponding to the 9 characteristic cells of OA immune infiltration. These 9 characteristic immune cells and their regression coefficients are shown in Table 2. Among the 9 characteristic immune cells, regulatory T cells, T follicular helper cells, Th1 cells, activated B cells, gamma delta T cells, effector memory CD4 T cells, CD56 bright natural killer cells, and CD56 dim natural killer cells were significantly infiltrated in OA, except for Th2 cells,

**TABLE 2:** Screening characteristic immune cells and their coefficients by the LASSO model.

Cell	coef
Regulatory T cell	0.6036
T follicular helper cell	0.0583
Type 1 T helper cell	0.2832
Activated B cell	0.8148
Gamma delta T cell	0.1434
Effector memory CD4 T cell	0.3602
CD56bright natural killer cell	0.2234
Type 2 T helper cell	−0.4255
CD56dim natural killer cell	0.5359

### 3.2 Identification of Imr-DGEs

**FIGURE 3:**
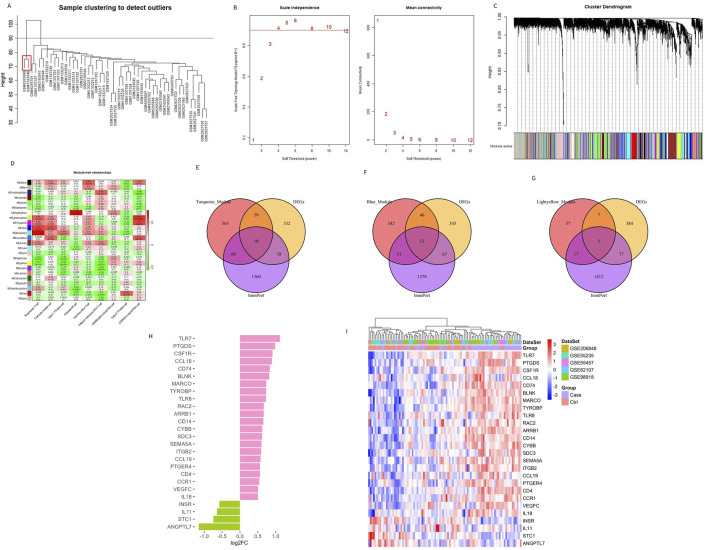
Identifying genes related to the immune microenvironment between OA and the control group. **(A)** Sample clustering and removal of outlier samples. **(B)** Soft threshold selection between OA and the control group. **(C)** A total of 471 DEGs in different co-expression modules. **(D)** Correlation heat map between each module and characteristic immune cells. **(E–G)** Genes from the turquoise, blue, and light yellow modules were intersected with DEGs and immune-related genes from the ImmPort database. **(H, I)** The bar chart and heat map display the differences in genes related to the differential immune microenvironments between patients with OA and controls.

Traditional DEG screening methods may overlook crucial factors, particularly in complex diseases like OA. To address this, we applied WGCNA to the combined dataset, focusing on identifying core gene modules associated with the nine characteristic immune cells identified in OA patients. During the clustering process, two outlier samples (highlighted in red boxes in Figure 3A) were excluded to ensure more accurate module detection. We optimized the scale-free topological network by setting the soft threshold to 4, as determined by the PickSoftThreshold function, ensuring robust network connectivity (Figure 3B). The hierarchical clustering algorithm then divided the dataset into 24 distinct gene modules, each represented by a different color (Figures 3C, D). Using the screening criteria for DEGs, we identified 471 DEGs in the combined dataset, with 233 downregulated and 238 upregulated genes (Supplementary Table S2). To further refine our analysis, we intersected these module genes with the ImmPort database, focusing on immune-related genes. This intersection revealed 26 Imr-genes across the turquoise, light yellow, and blue modules (10, 3, and 13 genes, respectively; Figures 3E–G). Further analysis revealed that in OA patients, the expression levels of *INSR, IL11, STC1,* and *ANGPTL7* were significantly lower than in control samples, while the other 22 genes were significantly upregulated (Figure 3H). Among these upregulated genes, *TLR7, PTGDS, CCL18, CCL19, BLNK, MARCO, IL18,* and *VEGFC* were particularly noteworthy due to their roles in inflammation and immune response (Figure 3I). These genes are integral to the formation and maintenance of the OA immune microenvironment, highlighting their potential as biomarkers or therapeutic targets in OA.

### 3.3 Correlation and functional enrichment of Imr-DEGs

**FIGURE 4:**
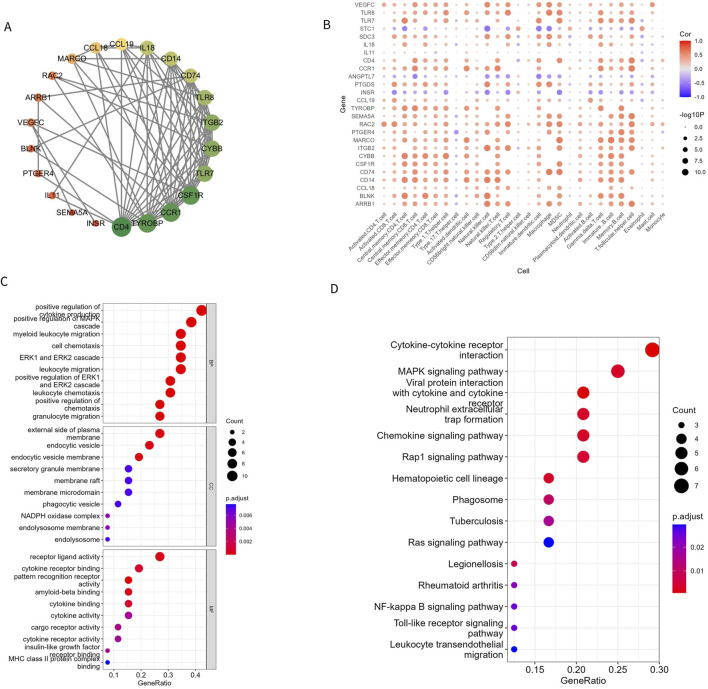
Imr-DEG interaction and functional enrichment. **(A)** A protein interaction network of 26 Imr-DEGs; each node represents a protein. If an interaction exists between two proteins, they are connected by a line. The larger the node, the more genes interact with it. **(B)** The correlation between 26 Imr-DEGs and 28 immune cells. **(C, D)** GO and KEGG functional enrichment bubble diagrams.

To understand the relationship between the 26 Imr-DEGs, we explored their interactions using the STRING database (Figure 4A). This analysis revealed a complex network of interactions among the Imr-DEGs, indicating their potential cooperative roles in the immune response within the OA microenvironment. Further correlation analysis between Imr-DEGs and 28 immune cell types highlighted significant associations, with many of these genes showing strong correlations with specific immune cells (Figure 4B). For example, genes such as *TLR7, CCL19,* and *IL18* were notably correlated with immune cells involved in inflammation and immune regulation, underscoring their roles in OA pathogenesis. GO functional enrichment analysis (Supplementary Table S3) provided deeper insights into the biological processes, cellular components, and molecular functions associated with these Imr-DEGs (Figure 4C). The most enriched biological processes included positive regulation of cytokine production, myeloid leukocyte migration, and cell chemotaxis, all of which are crucial for immune cell recruitment and activation in the OA environment. The cellular components mainly involved were the external side of the plasma membrane, endocytic vesicles, and endocytic vesicle membranes, which are key locations for immune signaling and antigen processing. On the molecular function level, pattern recognition receptor activity, receptor-ligand activity, and amyloid-beta binding were significantly enriched, suggesting a role for these genes in recognizing and responding to inflammatory signals. KEGG pathway enrichment analysis (Supplementary Table S4) further emphasized the involvement of these genes in critical immune-related pathways, including cytokine-cytokine receptor interaction, viral protein interactions with cytokine and cytokine receptors, and the MAPK signaling pathway (Figure 4D). These pathways are essential for mediating inflammatory responses and could drive the chronic inflammation observed in OA. The enrichment of these specific biological processes and pathways in both GO and KEGG analyses suggests that the Imr-DEGs play a significant role in orchestrating the immune response in OA, making them potential targets for therapeutic intervention.

### 3.4 Development and evaluation of machine learning model

**FIGURE 5:**
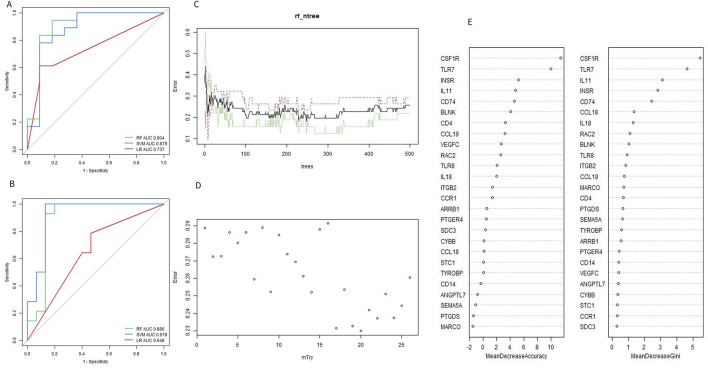
Evaluation of machine learning model. **(A, B)** ROC curves of the machine learning model for training set and validation set. **(C)** Selecting of the optimal number of binary tree variables (mtry value). **(D)** Selection of the optimal number of decision trees (ntree value). **(E)** Accuracy value and Gini value of Imr-DEGs.

To identify the optimal machine learning model for predicting OA, 95 samples from the combined datasets (49 OA and 46 controls) were randomly divided into a training cohort (70%) and a test cohort (30%). The training cohort consisted of 34 OA samples and 32 controls, while the test cohort included 15 OA samples and 14 controls. The expression profiles of 26 Imr-DEGs were selected as input variables to establish three machine learning models: RF, SVM, and LR. The AUC values for the RF, SVM, and LR models in the training set were 0.904, 0.879, and 0.737, respectively (Figure 5A). In the validation set, the AUC values were 0.886, 0.919, and 0.648, respectively (Figure 5B). The SVM model achieved the highest AUC in the test set, demonstrating strong predictive power. However, the RF model exhibited consistently high performance across both the training and test sets, making it the preferred model due to its balance of accuracy and robustness. The optimal performance of the RF model was achieved by combining the training and verification set results (Supplementary Table S5). Therefore, the RF model was selected for subsequent optimal feature screening. To optimize the RF model, the error rate was minimized when the mtry value was set to 17, and the model’s performance stabilized with an ntree value greater than 300 (Figures 5C, D). Finally, the RF model identified 9 genes with a Gini coefficient greater than 1 as the most important features (Figure 5E), including *BLNK, CCL18, CD74, CSF1R, RAC2, INSR, IL11, IL18,* and *TLR7*. These genes were considered key hub genes within the OA immune microenvironment, potentially serving as crucial biomarkers for OA diagnosis and progression.

### 3.5 Selection and verification of characteristic genes

**FIGURE 6:**
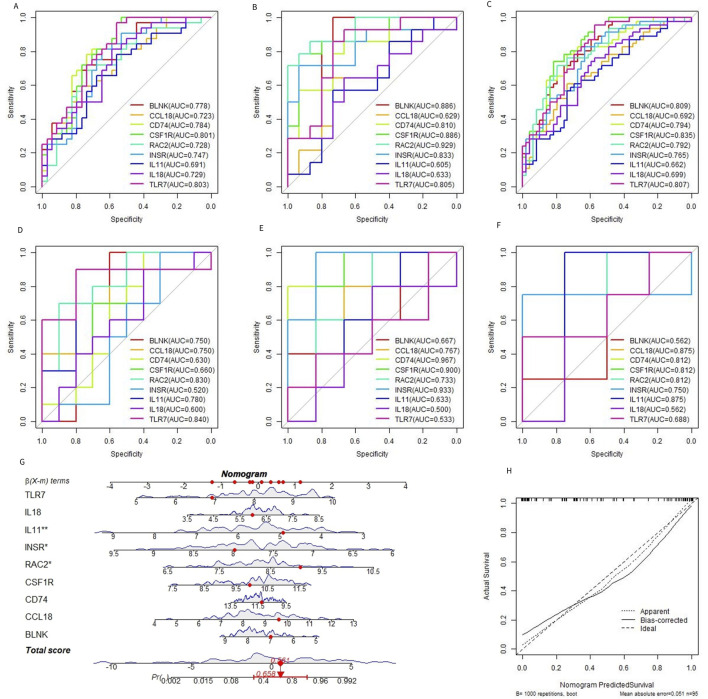
Diagnostic efficacy of characteristic genes. **(A–C)** ROC curves of each characteristic gene in the training set, verification set, and merged dataset. **(D–F)** ROC curves of characteristic genes in external datasets GSE117999, GSE169077, and GSE178557. **(G)** OA prediction nomogram based on the characteristic gene. **(H)**.

Based on the 9 characteristic genes identified by the RF model, ROC curve analysis was used to evaluate the diagnostic ability of each gene in predicting OA. The performance was assessed using the training dataset, the test dataset, and the combined dataset, as shown in shown in Figures 6A–C. External dataset validation was performed using GSE117999, GSE169077, and GSE178557, further confirming the diagnostic accuracy of these genes (Figures 6D–F). In addition to evaluating each gene individually, the 9 genes were combined to construct a predictive tool for OA development, presented as a nomogram. The nomogram assigns a score to each feature variable based on its expression level, with the total score corresponding to the predicted risk of developing OA. As shown in Figure 6G, the total score ranged from 0 to 5, with the associated risk probability for OA ranging from 0.558 to 0.992. This finding indicates that the nomogram can provide a highly individualized risk assessment for OA, which could be valuable in clinical decision-making. Furthermore, the accuracy of the nomogram was validated using a calibration curve, which confirmed the model’s predictive reliability (Figure 6H). The calibration curve demonstrated that the predicted probabilities closely aligned with the actual observed outcomes, underscoring the nomogram’s utility as a diagnostic tool in clinical settings.

### 3.6 Identification of immune subtypes in patients with OA

**FIGURE 7:**
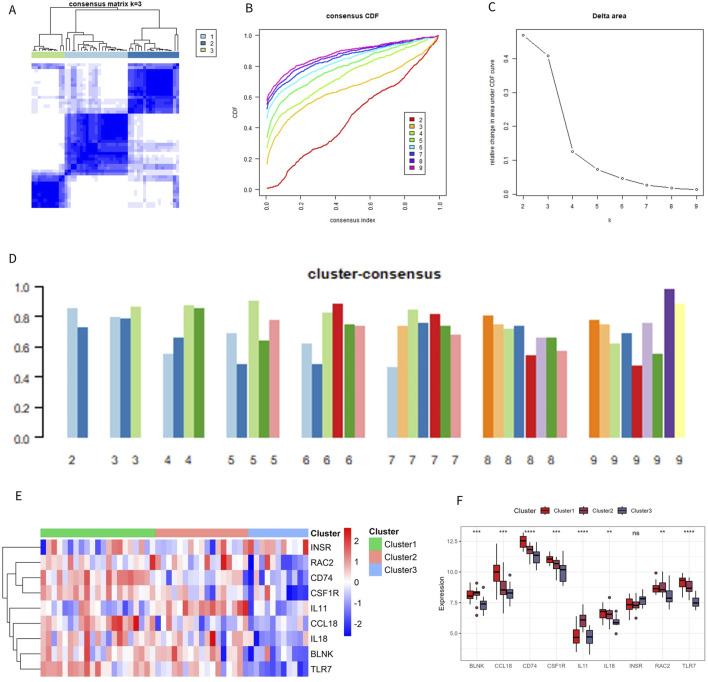
Identification of immune subtypes. **(A)** C onsensus clustering matrix at k = 3. **(B)** Consensus CDF curve for k = 2–9. **(C)** Change in the delta area curve of the CDF. **(D)** Consistency score for k = 2–9. **(E, F)** Heatmap and box plot of characteristic genes in different subtypes.

To clarify the expression pattern of OA and the immune microenvironment, 49 OA patients in the combined dataset were analyzed by consistent cluster analysis based on 9 characteristic genes. According to the consistent clustering results, including the Cumulative Distribution Function (CDF) diagram, area change of the CDF curve, and clustering score, k = 3 was selected as the optimal value, dividing the OA patients into three different subtypes: Cluster 1 (21 cases), Cluster 2 (17 cases), and Cluster 3 (11 cases) (Figures 7A–D). The expression patterns of the characteristic genes varied among the different subtypes. Except for *INSR* and *IL11*, the expression of the other genes in Cluster 1 and Cluster 2 was significantly higher than in Cluster 3 (Figures 7E). The distribution of consistently clustered samples in the different clusters is shown in Figures 7F.

### 3.7 Different immune characteristics of immune subtypes

**FIGURE 8:**
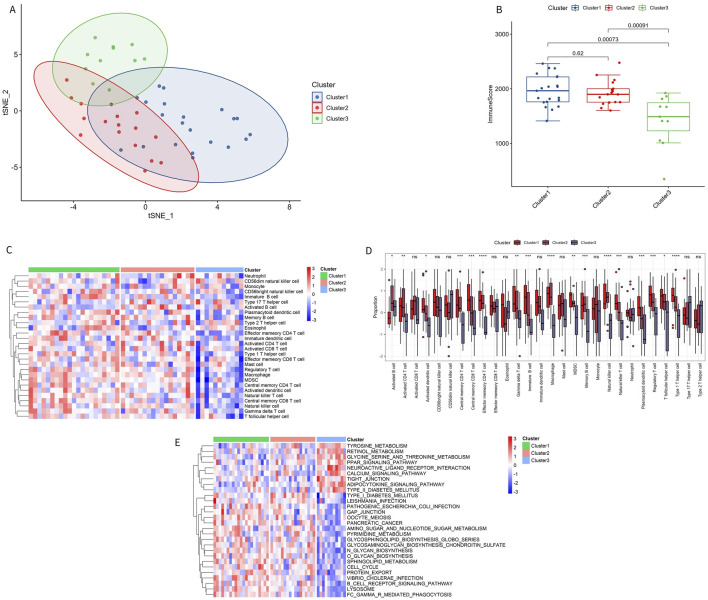
Immune characteristics of immune subtypes. **(A)** t-SNE analysis of different subtypes. **(B)** Comparison of immune scores across different subtypes. **(C, D)** Distribution of 28 types of immune cells in different subtypes. **(E)** GSVA pathway enrichment in different subtypes.

To better understand the biological and immunological differences of these immune subtypes and their relationship, tSNE analysis was conducted on the different subtypes, revealing greater overlap between Cluster 1 and Cluster 2, while Cluster 3 remained relatively independent (Figure 8A). In conjunction with the distribution of ImmuneScore among the subtypes, Cluster 3 exhibited distinct immune score characteristics compared to the other two groups, indicating that Cluster 3 had a unique expression pattern (Figure 8B).

Among the 28 immune subtypes, 17 immune cells showed significant differences. Some of these common immune cells, such as activated B cells, activated CD8 T cells, macrophages, natural killer cells, and regulatory T cells, play a significant role in the inflammatory immune response. The proportion of these immune cells with significant differences was higher in Cluster 1 and Cluster 2 than in Cluster 3 (Figures 8C, D). The enrichment pathways in Cluster 1 and Cluster 2 were consistent with the above results, with immune microenvironment-related pathways activated in these two subtypes, such as B-CELL-RECEPTOR-SIGNALING-PATHWAY and CELL-CYCLE (Figure 8E). The significant enrichment results of GSVA GO and KEGG are shown in Supplementary Tables S6, S7.

### 3.8 Relationship between characteristic genes and drugs

**FIGURE 9:**
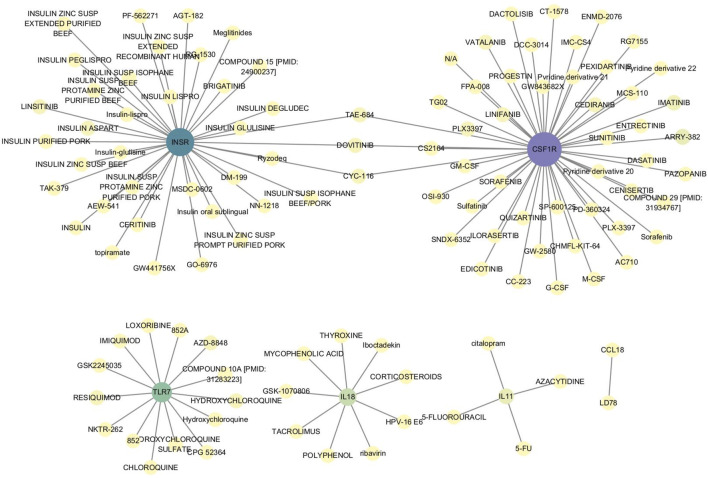
Interaction between characteristic genes and therapeutic drugs. Drug-gene interaction analysis was conducted through the DGIdb database to identify potential therapeutic targets for OA. Six characteristic genes—*CSF1R, INSR, TLR7, IL11, IL18,* and *CCL18*—showed interactions with various drugs, indicating their roles in modulating immune responses, inflammation, and cellular signaling pathways. These interactions suggest that these genes could be key targets for developing or repurposing drugs aimed at treating OA.

To further explore the role of these characteristic genes, the relationship between genes and drugs was investigated using the DGIdb database. Six characteristic genes—*CSF1R, INSR, TLR7, IL11, IL18,* and *CCL18—*were found to have drug interactions (Figure 9; Supplementary Table S8).

## 4 Discussion

**FIGURE 10:**
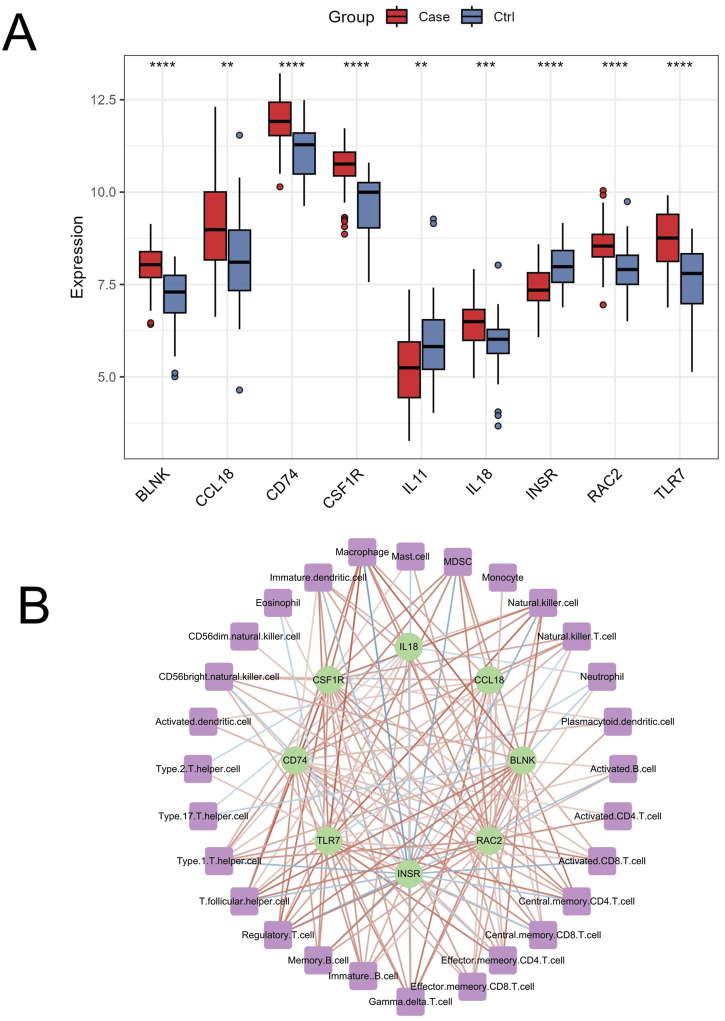
Differential expression and immune cell correlation of characteristic genes in the merged dataset. **(A)** Boxplots displaying the differential expression of the 9 characteristic genes (*BLNK, CCL18, CD74, CSF1R, RAC2, INSR, IL11, IL18,* and *TLR7*) between OA and normal control samples in the merged dataset. ∗∗*p* < 0.01, ∗∗∗∗*p* < 0.0001 **(B)** The correlation analysis between the 9 genes and immune cells, extracted from Figure 4B, shows significant positive and negative correlations (*p* < 0.05), with stronger associations represented by darker red (positive) and darker blue (negative) lines.

OA is the most common type of arthritis, leading to the irreversible destruction of articular cartilage and bone, and is one of the primary causes of physical disability. OA significantly impacts human health, resulting in a substantial economic burden on society (Katz et al., 2021). Although many scholars have extensively studied OA, there is still no method for early and effective diagnosis of the disease. The present study innovatively combined ssGSEA, WGCNA, bioinformatics, and machine learning algorithms to identify OA immune-related markers and assess the clinical risk of OA. We identified 9 genes, including *BLNK, CCL18, CD74, CSF1R, RAC2, INSR, IL11, IL18, and TLR7*, as characteristic biomarkers associated with OA immune infiltration. The differential expression of these genes in OA compared to normal controls in the merged dataset is shown in Figure 10A. *IL11* and *INSR* were significantly downregulated in OA, while the remaining genes were significantly upregulated. Furthermore, these genes were significantly correlated with various immune cells (Figure 10B). These findings highlight the crucial role of immune regulation in the pathogenesis of OA and suggest potential therapeutic targets for intervention.

In this study, 9 characteristic cells with significant differences were identified out of 28 immune cell subsets, most of which belonged to the lymphocyte lineage. Rossshirt et al. found significant immune cell infiltration, cell polarization, and increased expression of various cytokines in the synovium and synovium of patients with early OA(19). We hypothesized that regulatory T cells, T follicular helper cells, Th1 cells, activated B cells, gamma delta T cells, effector memory CD4 T cells, CD56 bright natural killer cells, significant infiltration of CD56 dim natural killer cells, and a reduction of Th2 cells may be related to the occurrence and development of OA.

There is sufficient research evidence to support the above conjectures. Li et al. found that an imbalance of regulatory T cells in OA leads to an abnormal increase in IL-2, which in turn causes the degeneration of articular cartilage (Li X. et al., 2022). Previous studies have also shown that macrophages in the synovium can promote the proliferation and differentiation of T follicular helper cells in joint tissues through the OX40/OX40L axis, thereby contributing to the pathogenesis of OA (Cai et al., 2022). According to Kalaitzoglou et al., OA is associated with abnormal innate immune inflammatory response, characterized by significant infiltration of CD4 T cells, which eventually stimulates the polarization of Th1 cells and exacerbates the release of immune response cytokines (Kalaitzoglou et al., 2017; Zhao, 2021). Additionally, scholars have found that end-stage OA is characterized by characteristic CD1T cell infiltration, Th1 cell polarization, and a large amount of release of various cytokines, which may be one of the important reasons for the irreversible deterioration of OA (Rosshirt et al., 2019; Platzer et al., 2022). Interestingly, some studies have shown that TH1 and Th2 cells contribute simultaneously to the progression of OA; however, this differs from the predicted results of this study (Rosshirt et al., 2021; Nees et al., 2020). The relevant research evidence is insufficient, partly because the two types of T helper cells are in a constant state of dynamic changes, and their proportions vary at different stages of OA development (Burt et al., 2022). The expression of B cells in OA tissues also fully illustrates their role. Xie et al. found that B cells in OA synovium exhibit stronger proliferation and differentiation capabilities and can continue to produce antibodies, leading to tissue damage (Xie et al., 2023). The results of our analysis, combined with evidence from the literature mentioned above, suggest that regulatory T cells, T follicular helper cells, Th1 cells, activated B cells, gamma delta T cells, and effector memory CD4 T cells play important roles in OA and should be the focus of further research. However, gamma delta T cells have so far been identified only in rheumatoid joints, where they significantly promote disease progression (Mo et al., 2017). Further experimental data are needed to investigate the role of CD56 bright natural killer cells and CD56 dim natural killer cells in OA. From an immune response perspective, evaluating the diversity of cells and gene expression in the OA immune microenvironment is of great value in revealing the mechanism of OA and predicting the risk of its occurrence.

Using the ImmPort database, we found that among the identified Imr-DEGs, the expression levels of 22 genes were significantly higher in OA than in controls. Among these genes, *TLR7, PTGDS, CCL, BLNK, MARCO, IL18,* and *VEGFC* are involved in the immune-inflammatory response of OA, promoting its development (Hoshikawa et al., 2020; Tu et al., 2023; Liao et al., 2022; Cheng et al., 2021). KEGG analysis revealed that Imr-DEGs were enriched in important pathways related to immunity and inflammation, such as the cytokine-cytokine receptor interaction and the MAPK signaling pathway. The MAPK signaling pathway is closely related to inflammatory cartilage destruction and imbalances in cartilage extracellular matrix metabolism (Jiang et al., 2023; Zhao C. et al., 2023; Ni et al., 2023). The cytokine-cytokine receptor interaction is readily triggered in the late stages of injury and is significantly enriched in models leading to chondrocyte inflammation via tumor necrosis factor-alpha stimulation (Wang et al., 2020). GO enrichment analysis also detected biological processes related to these Imr-DEGs, including immune responses such as the positive regulation of cytokine production, cell chemotaxis, and myeloid leukocyte migration. OA is a chronic inflammatory disease closely related to immune cell infiltration. Immune cells in the OA synovium synthesize and release various cytokines and chemokines, which play an important role in cartilage matrix degradation (Bernardini et al., 2017). Furthermore, cytokine and chemokine action are among the most important factors in the pathogenesis of rheumatoid arthritis and OA, two inflammatory joint diseases. The results of this study confirmed that immune-related reactions are an important factor in the occurrence and progression of OA. Future studies should further explore the different immune subtypes and immune characteristics of OA.

To better identify the different immune subtypes and characteristics of OA in the data set, we screened 9 characteristic genes (*BLNK, CCL18, CD74, CSF1R, RAC2, INSR, IL11, IL18,* and *TLR7*) using the optimal machine learning algorithm. Currently, 7 of these 9 genes have been extensively studied. According to previous research, *BLNK* promotes inflammation and plays a role in the progression of OA (Cheng et al., 2021; Li et al., 2020). *CCL18*, a member of the chemokine family, mainly targets lymphocytes and immature dendritic cells and is involved in maintaining immune system homeostasis under physiological conditions (Cardoso et al., 2021). *CCL18* has also been identified by Mertens and others as a biomarker of localized scleroderma disease activity. In this study, *CCL18* was studied as a characteristic gene of OA for the first time (Mertens et al., 2019). CD74 (MHC class II invariant chain, Ii) is a non-polymorphic type II transmembrane glycoprotein that acts as both an MHC II chaperone and a receptor of macrophage migration inhibitory factor, regulating the development and movement of T and B cells (Schröder, 2016). *CD74* also plays a significant role in many inflammatory diseases, suggesting a potential relationship with OA (Su et al., 2017). Colony-stimulating factor-1 receptor (CSF-1R) regulates the proliferation, migration, and activation of monocytes. By analyzing synovial samples from OA and control groups, Myew-Ling showed that the expression of CSF-1R was significantly increased in the OA group. Blocking CSF-1R expression can reduce cartilage injury, bone erosion, and systemic bone loss (Toh et al., 2014). IL11 and IL18 are cytokines involved in hematopoiesis, cancer metastasis, and inflammation. Their expression has been confirmed to be significantly increased in OA tissues, consistent with the findings of our study (Lisignoli et al., 2000; Paolucci et al., 2023). Toll-like receptor 7 (*TLR7*) is another gene associated with the risk of OA (Xi et al., 2022). Although research on *RAC2* and *INSR* genes in OA is still limited, we evaluated their diagnostic potential for OA using ROC curves. However, further research is needed to establish a theoretical basis for these findings. Based on the 9 characteristic genes, we also constructed an OA prediction tool (nomogram), and the correction curve confirmed its effectiveness in risk prediction.

The present study combines multiple advanced methodologies, including ssGSEA, WGCNA, bioinformatics, and machine learning algorithms, to explore the immune microenvironment in OA. While these approaches have significantly contributed to identifying potential biomarkers and understanding OA pathogenesis, they also highlight the need for more integrative and comprehensive studies in the future. Current approaches often face challenges due to the complexity of OA, including its heterogeneity and the dynamic nature of immune responses. Future research should focus on validating these findings in larger, more diverse cohorts, and on developing translational tools that can bridge the gap between molecular discoveries and clinical applications. Additionally, emerging techniques like single-cell RNA sequencing and spatial transcriptomics could provide even deeper insights into the cellular interactions within the OA microenvironment, potentially leading to the development of more targeted therapies.

There are still some limitations in the present study. First, due to the complexity and diversity of OA diseases, the samples selected in this study come from multiple GEO data sets, so they cannot fully represent all types of OA. Additionally, the clinical parameters such as stage, age, and sex of the patients studied were not available, which are crucial factors given the multifactorial nature of OA, and it is unclear if the datasets belong to the same stage of OA, which may necessitate reanalysis with stage-specific OA in mind. Furthermore, This study only investigated the correlation between OA and the immune microenvironment and the occurrence of risk prediction based on markers, while the mechanism has not been further studied. Lastly, wet lab experiments need to be conducted to validate the expression of the identified biomarkers, and including more OA datasets in future studies will help refine the findings and support their translation into clinical applications.

## 5 Conclusion

The study found significant differences in immune cell infiltration between OA patients and controls, identifying distinct immune subtypes. It also highlighted key immune-related genes strongly associated with OA. These genes and immune cells could be valuable for assessing disease progression or treatment effectiveness. Furthermore, machine learning models, especially the RF model, showed high accuracy in predicting OA. These biomarkers could be integrated into clinical tools for OA diagnosis and prognosis, supporting more personalized treatment. However, due to the lack of prospective clinical studies, further validation is needed before these biomarkers can be routinely used in practice.

## Data Availability

The original contributions presented in the study are included in the article/Supplementary Material, further inquiries can be directed to the corresponding author.
