# Supplementary material for: Identification of immune microenvironment subtypes and clinical risk biomarkers for osteoarthritis based on a machine learning model
Source: Front Mol Biosci. 2024 Oct 17;11:1376793. doi: 10.3389/fmolb.2024.1376793 (PMC11524973; doi:10.3389/fmolb.2024.1376793)
Supplement: Supplementary file 5 [file DataSheet5.DOCX]

Table S5 RF Model performance metrics for training and validation set.

| **Metric** | **Training Set** | **Validation Set** |
| --- | --- | --- |
| Accuracy | 100% | 83% |
| Sensitivity | 100% | 79% |
| Specificity | 100% | 87% |
